# Supplementary material for: Improving the Corrosion Protection of Poly(phenylene methylene) Coatings by Side Chain Engineering: The Case of Methoxy-Substituted Copolymers
Source: Int J Mol Sci. 2022 Dec 17;23(24):16103. doi: 10.3390/ijms232416103 (PMC9784788; doi:10.3390/ijms232416103)
Supplement: Supplementary file 1 [file ijms-23-16103-s001.zip › ijms-2032934-supplementary.pdf]

Supporting materials

Improving the corrosion protection of poly(phenylene methylene) coatings by side chain engineering: the case of methoxy-substituted copolymers

Marco F. D'Elia*, Mirko Magni*, Stefano P. M. Trasatti, Markus Niederberger, Walter R. Caseri

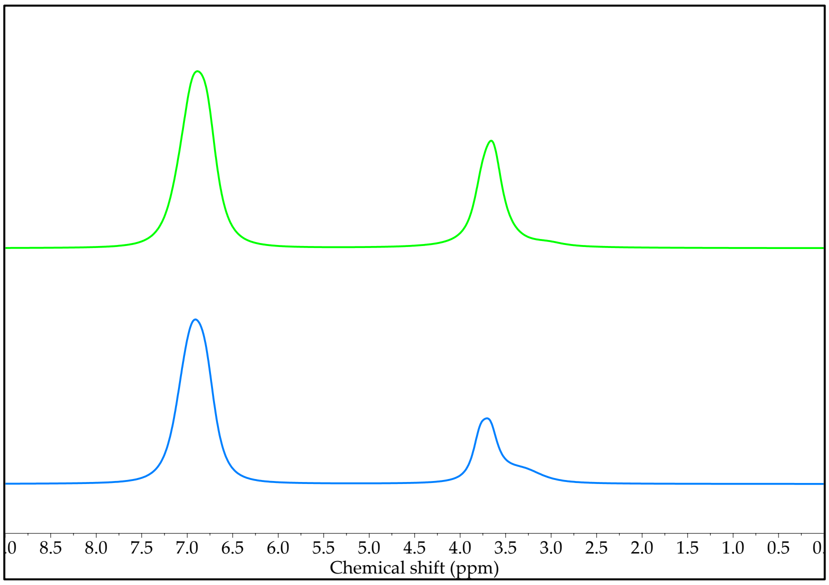

Figure S1: ^{1}H-NMR ($CDCl_3$) of PPM copolymers containing 4.6% mol/mol (green) and 9% mol/mol (blue) of methoxy side chains.

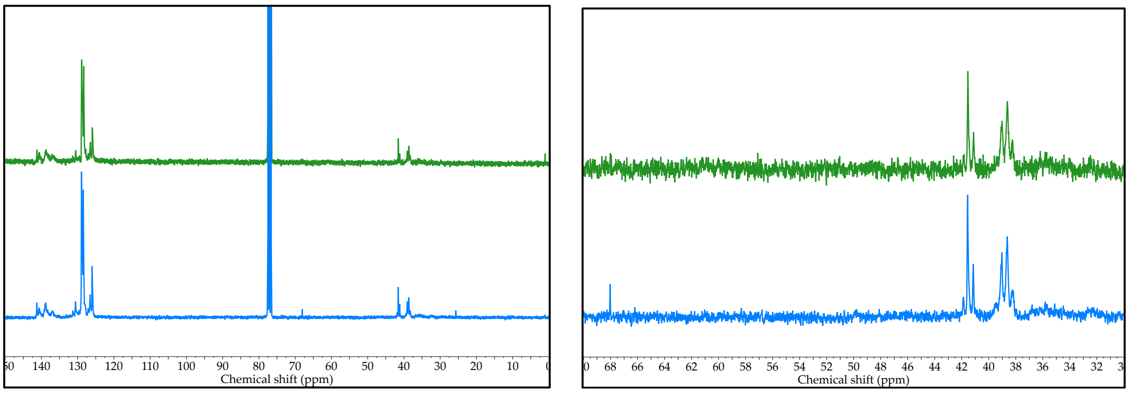

Figure S2: ^{13}C-NMR ($CDCl_3$) of PPM copolymers containing 4.6% mol/mol (green) and 9% mol/mol (blue) of methoxy side chains. Left: full spectra; right: magnification of the range from 70 to 30 ppm.

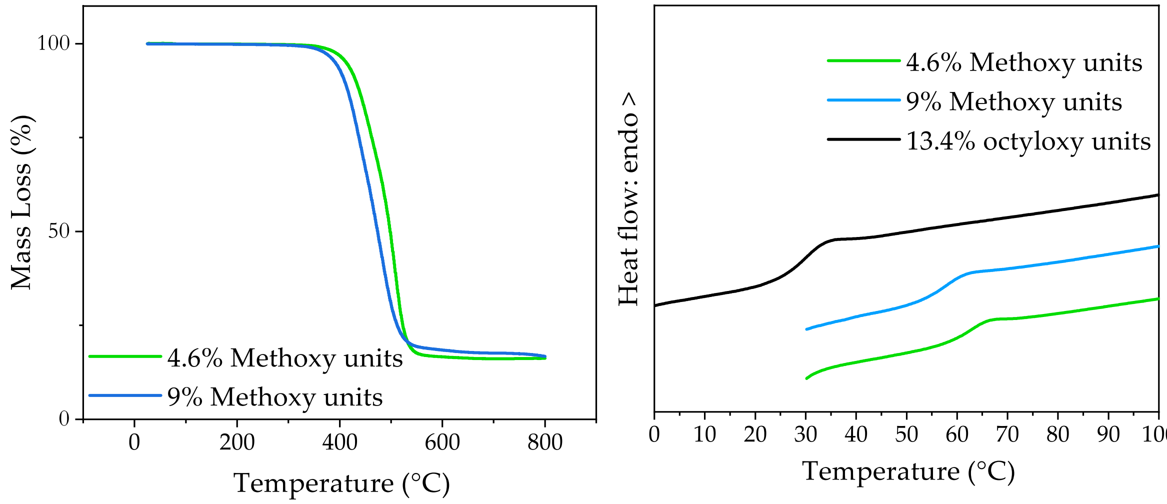

Figure S3: TGA (left) and DCS (right) traces of PPM copolymer containing 4.6 and 9.0 % mol/mol methoxy side chains. For sake of comparison, the DSC investigation on PPM copolymer containing octyloxy side chains (13.4 % mol/mol) is also reported.

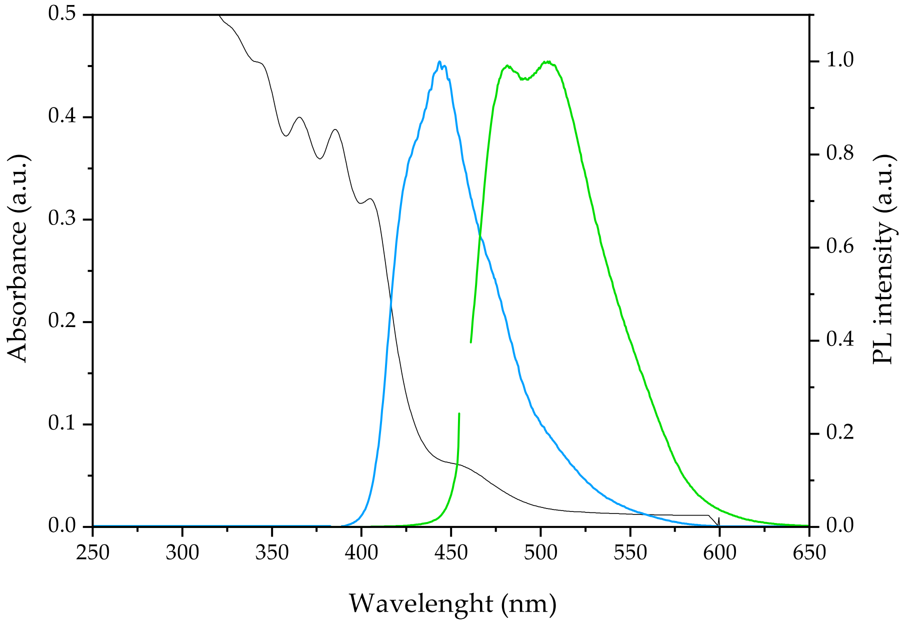

Figure S4: UV-vis absorption (black line) and photoluminescence emission (colored lines) spectra of a 0.5 wt.% THF solution of PPM copolymer containing 9.0 % mol/mol methoxy side chains. The blue line corresponds to the high-energy emissive phase (blue phase; λ_{exc}= 386 nm), while the green line corresponds to the lower energy emissive phase (green phase; λ_{exc}= 455 nm) of the polymer.

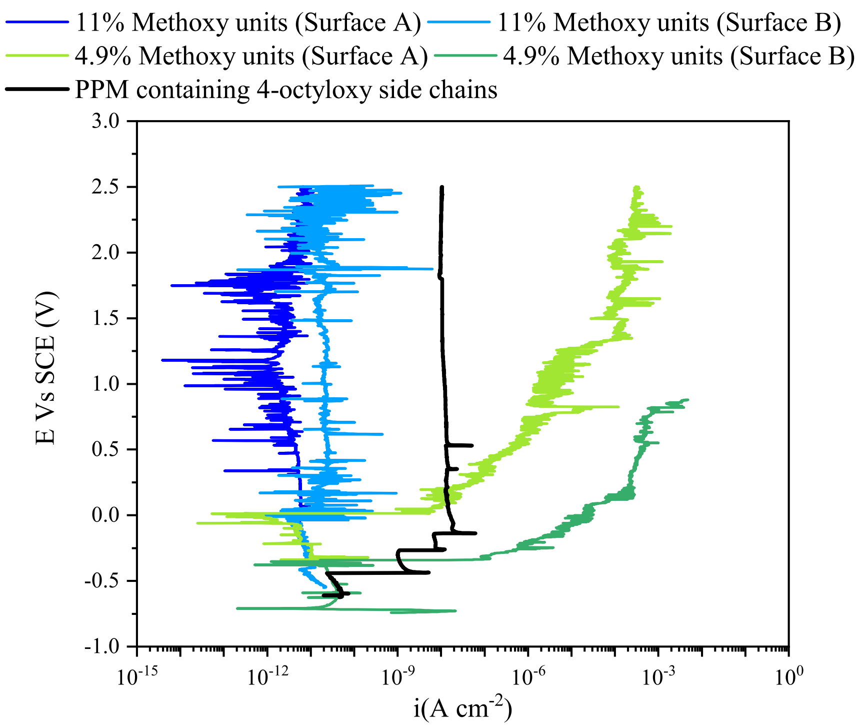

Figure S5: Comparison of potentiodynamic anodic polarization curves obtained for replicates of AA2024 samples coated by co-PPM 4.6% (green tones) and co-PPM9.0% (blue tone) in 3.5 %w/v NaCl solution. Potential scan rate: 10 mV/min. For sake of comparison, a representative curve for the best formulation of PPM copolymer containing octyloxy side chains is also reported (black line) [12].

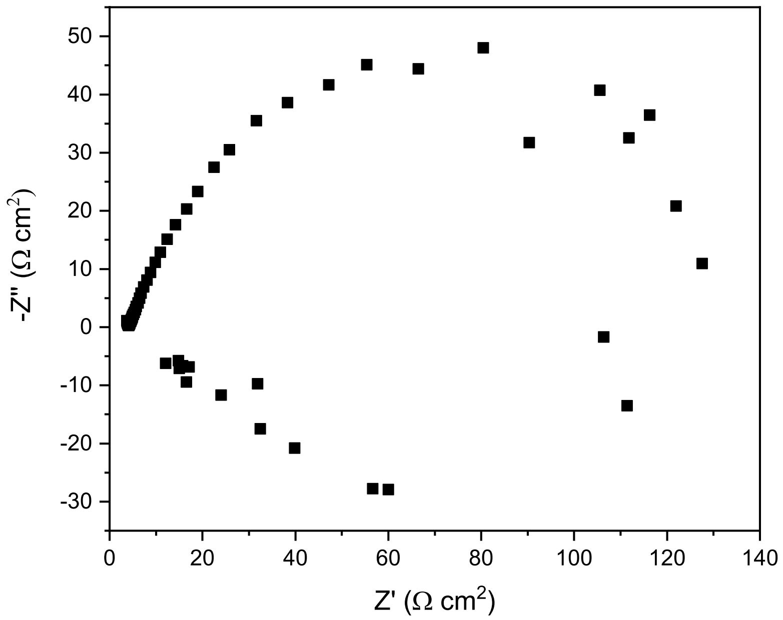

Figure S6: Nyquist plot of the first EIS spectra recorded on artificially damaged AA2024 sample coated by co-PPM 9.0% showing inductive loop.

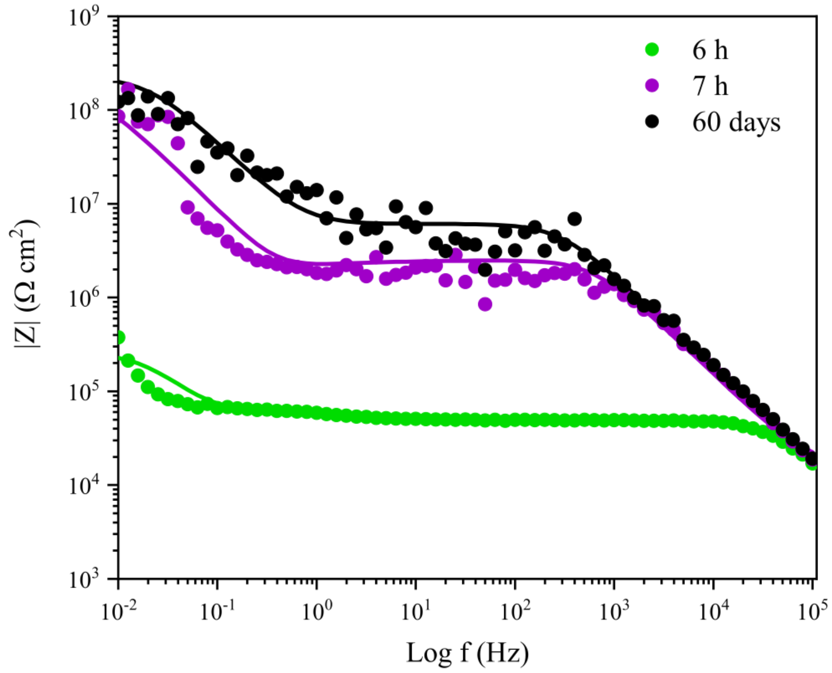

Figure S7: Representative Bode modulus spectra (dots: experimental data; lines: fitted spectra) recorded during the long-time monitoring of an artificially damaged AA2024 sample coated by co-PPM 9.0%.

Synthetic procedure S1: Synthesis of copolymers of phenylene methylene and 4-methoxyphenylene methylene

Copolymerization was carried out by adding 4-methoxybenzyl chloride under nitrogen atmosphere to 15 g (0.12 mol) destabilized benzyl chloride in a 100 mL three-neck flask containing 0.07 g (0.1 mmol) of bismuth (III) trifluoromethansulfonate. Accordingly, 4-methoxybenzyl chloride was in differen concentrations (mol/mol) respect to benzyl chloride (5% and 10%) adding 0.006 mol and 0.013 mol, respectively. The mixture was then heated up to 60 °C under a constant nitrogen flow of 0.4–0.5 mL min^{-1} to avoid the produced HCl from reaction environment. Because the increase of the viscosity during polymerization, after 3 h the temperature was risen up to 120 °C for 3 h and subsequently to 180 °C for 17 h in order to guarantee a constant stir. In process controls were performed by analyzing with ^{1}H-NMR samples aliquot from the reaction mixture over the reaction time in order to assess the completion of the reaction. Afterwards, the product was cooled down to room temperature and dissolved in 10 mL THF. The THF solution was then poured into 400 mL methanol under vigorous stirring for 4 h. The obtained powder was filtered through a cellulose filter and dried under vacuum (10^{-2} mbar) for 12 h. As established by ^{1}H-NMR spectra of the product the copolymers containing 4.6% (7.4 g) and 9 % mol/mol (9 g) of methoxy repeat units were obtained.
